# GPCR signaling systems facilitate precision interventions for multi-oncology therapy

**DOI:** 10.3389/fsysb.2026.1940022

**Published:** 2026-09-22

**Authors:** Ashley Del Pozo, Mia Pardo, Tabitha Boeringer, Carter J. Craig, Ramzia Sorathia, Madeline Millican, Stuart Maudsley

**Affiliations:** Receptor Biology Lab, Department of Drug Discovery, H. Lee Moffitt Cancer Center and Research Institute, Tampa, FL, United States

**Keywords:** adaptive therapy, biased signaling, CtRNA, digital twins, GPCR, integrative genomics, liquid biopsy, multi-omics

## Abstract

Precision oncology has undergone a transformative evolution from static genomic profiling toward dynamic, multi-omics frameworks capable of capturing tumor heterogeneity and adaptive resistance in real time. Central to this paradigm shift is the emerging recognition that G protein-coupled receptors (GPCRs) constitute a vastly underexploited axis of oncogenic signaling, tumor microenvironment (TME) modulation, and non-genetic adaptive resistance. Here we delineate the effective transition to GPCR-focused precision oncology, emphasizing the integrated deployment of next-generation sequencing (NGS), single-cell transcriptomics, expression-based liquid biopsies (ctRNA, exosomal miRNA, circulating tumor cells), and AI-driven quantitative systems network modeling to interrogate GPCR signaling dynamics across solid and hematologic malignancies. GPCRs, comprising approximately 800 human receptors with extensive pharmacological plasticity, encompassing biased agonism, allosteric modulation, receptor heterodimerization, and ligand-independent signaling, serve as critical integrators of extracellular cues and intracellular transcriptional programs. Their overexpression, mutation, and epigenetic dysregulation in multiple cancer types positions them as both prognostic biomarkers and actionable therapeutic targets. We discuss recent advances in integrative genomic platforms, liquid biopsy evolution, case studies of GPCR-driven tumor evolution, and cutting-edge therapeutic modalities, including PROTACs (Proteolysis Targeting Chimeras), biased ligands, RNA therapeutics, and digital twin modeling, to articulate a forward-looking vision for GPCR-centric precision oncology. By harnessing real-time tumor monitoring and AI-optimized adaptive therapies, this framework promises to overcome the limitations of static genomic approaches, enabling mid-treatment recalibration to combat resistance and improve durable survival in heterogeneous solid tumors.

## 1 Introduction

### 1.1 Precision cancer interventions

The concept of precision oncology interventions was galvanized by the advent of next-generation sequencing (NGS) and companion liquid biopsy diagnostics, which together enabled clinicians to match targeted therapies to actionable driver mutations in individual patients. Landmark regulatory approvals, including Guardant360 and FoundationOne Liquid CDx, established comprehensive platforms capable of profiling over 300 cancer-associated genes alongside key genomic features such as microsatellite instability (MSI) and tumor mutational burden (TMB), thereby informing therapy selection in non-small cell lung cancer (NSCLC) and numerous other solid tumor types (Masina and Caldas, 2024; Gulati et al., 2026). Celebrated examples of this era include EGFR-directed tyrosine kinase inhibitors in NSCLC, BRAF/MEK inhibitors in melanoma, and PARP inhibitors in BRCA-mutant ovarian cancer, each representing paradigmatic instances where genomic precision translated into meaningful clinical benefit.

Despite these advances, early iterations of precision oncolgy were fundamentally constrained by their primary reliance on static genomic snapshots. While mutation-based profiling can identify initial therapeutic vulnerabilities, it often captures poorly the dynamic biological processes, including epigenetic reprogramming, transcriptional plasticity, and microenvironmental adaptation, that drive-acquired resistance and tumor evolution under treatment pressure (Vasconcellos et al., 2018). The proportion of patients harboring genuinely actionable mutations amenable to approved targeted agents remains a minority across most cancer types, emphasizing the need to expand the molecular landscape of precision oncology beyond somatic DNA alterations alone.

### 1.2 Flaws in static genomic approaches

Intratumoral heterogeneity represents a fundamental biological challenge to static profiling strategies (Zhu et al., 2021). Tumors are not monolithic molecular entities, but represent highly nuanced, dynamic ecosystems comprised of genetically and epigenetically distinct subclones that evolve under selective therapeutic pressures (Mubin et al., 2026; Espinosa-Sánchez and Carnero, 2026). Traditional NGS panels capture a single temporospatial biopsy, thereby missing the subclonal architecture, spatial transcriptomic variability, and real-time adaptive reprogramming that collectively orchestrate resistance (Sriram et al., 2019). In practical terms, this manifests as initial treatment responses followed by relapse driven by pre-existing or acquired resistant clones.

Compounding this limitation is the systematic underrepresentation of GPCRs in standard oncology panels, despite mounting evidence for their overexpression, mutation, and functional dysregulation across cancers (Bar-Shavit et al., 2016; Wu et al., 2019). CXCR4, for example, is overexpressed in over 23 different human cancers and plays a pivotal role in metastatic dissemination and immune evasion (Furusato et al., 2010). Similarly, GPRC5A drives metastatic colonization in lung and esophageal cancers, while proton-sensing GPCRs like GPR65 facilitate metabolic adaptation to the acidic TME (Ahn and Kaipparettu, 2025; Zhou et al., 2024). These receptors operate largely through non-mutational mechanisms, ligand gradient alterations, transcriptional upregulation, epigenetic reactivation, which escape detection by DNA-centric panels.

Access and implementation disparities further amplify these gaps. Analyses of real-world precision oncology deployment, such as in the Brazilian public healthcare system (SUS) for NSCLC, illustrate how structural limitations constrain the translation of genomic discoveries into equitable clinical practice, with many patients unable to access advanced molecular diagnostics or matched therapies (Schneider et al., 2025). Addressing these disparities requires not only technological innovation but also deliberate health system reform.

### 1.3 Emergence of the second iteration of cancer precision pharmacology

The conceptual advance of further iterations in the field of precision oncology relied upon the functional shift from a static, mutation-centric paradigm toward a dynamic, multi-omics, expression-guided framework capable of tracking tumor evolution in real time (Gulati et al., 2026). Central to this evolution was the integration of expression-based liquid biopsy modalities, circulating tumor RNA (ctRNA), exosomal microRNA, and cell-free RNA, which capture functional signaling states rather than merely genomic alterations. These analytes enable longitudinal monitoring of GPCR expression dynamics, biased signaling transitions, and transcriptional rewiring that presage resistance before it manifests clinically (Maudsley et al., 2022).

The approximately 800 human GPCRs represent an expansive signaling network uniquely suited to serve as sensors integrating multi-omics information streams (Boeringer et al., 2026; Craig et al., 2026). Their pharmacological complexity encompassing G protein-biased agonism, β-arrestin-mediated signaling, allosteric modulation, and receptor heterodimerization, confers both the plasticity required for adaptive signaling and the molecular specificity amenable to precision targeting (Leysen et al., 2021; McCorvy et al., 2018; Lappano and Maggiolini, 2012). Emerging tools such as AI-driven network models (e.g., TMO-Net), digital twin platforms, and tumor-agnostic GPCR profiling are coalescing to enable patient-specific simulations of GPCR signaling dynamics, offering a route toward truly individualized adaptive therapies (Boeringer et al., 2025; Liu et al., 2025; Wang et al., 2024).

Thus, here we posit that the integration of multi-omics genomic profiling with liquid biopsy-guided GPCR monitoring will fundamentally transform the precision oncology landscape, enabling proactive relapse prevention and rational design of combination regimens. (a dimension-by-dimension contrast with the conventional mutation/ctDNA-centric paradigm is provided in Table 1). We synthesize recent advances encompassing integrative genomic platforms, liquid biopsy innovations, mechanistic case studies of GPCR-driven tumor evolution, and next-generation therapeutic modalities including biased ligands, PROTACs, CRISPR-based discovery platforms, and AI-optimized digital twins. Our aim is to provide a comprehensive, modern perspective on GPCR-centric adaptive oncology strategies that can be incorporated into clinical workflows to extend durable therapeutic responses across diverse cancer indications (Figure 1).

**TABLE 1:** GPCR-directed therapeutic modalities in oncology.

Modality	Mechanism	Example GPCR target(s)	Key advantage	Manuscript section
Biased ligands	Selectively engage anti-tumor signaling arms (e.g., β-arrestin) while avoiding pro-tumorigenic G protein pathways	Aminergic GPCRs; FZD7	Functional selectivity reduces on-target toxicity for chronic dosing	4.4, 5.2
PROTACs	Recruit E3 ubiquitin ligases to degrade overexpressed receptors rather than block signaling	Scaffolding/PPI-dependent GPCRs	Addresses receptors with no druggable orthosteric/allosteric pocket	5.1
RNA therapeutics (ASOs, siRNA, mRNA, CRISPR modulators)	Sequence-specific modulation of GPCR expression, independent of protein structure	Conserved GPCR signaling hubs across cancer types	Reaches “undruggable” GPCRs lacking small-molecule binding sites	5.3
CRISPR-based discovery platforms (e.g., DCyFIR)	Multiplexed, high-throughput profiling of receptor activation across signaling readouts	Pathway-selective ligand discovery	Accelerates identification of biased/selective compounds	5.1
GPCR-targeted CAR-T/antibodies	AI-optimized antibodies against surface-expressed GPCRs for immune cell engineering	Novel CAR-T target GPCRs	Extends immunotherapy to GPCR-overexpressing tumors	2.3
Small-molecule antagonists	Competitive/allosteric blockade of receptor-ligand engagement	CXCR4 (AMD3100), FZD7 (cycloartobiloxanthone)	Established pharmacology, clinically precedented	4.1–4.3
Digital twin-guided regimen selection	Computational modeling of patient-specific GPCR network behavior from longitudinal multi-omics data	Patient-specific signaling network	Enables adaptive, mid-treatment recalibration	5.2, 6.3

**FIGURE 1:**
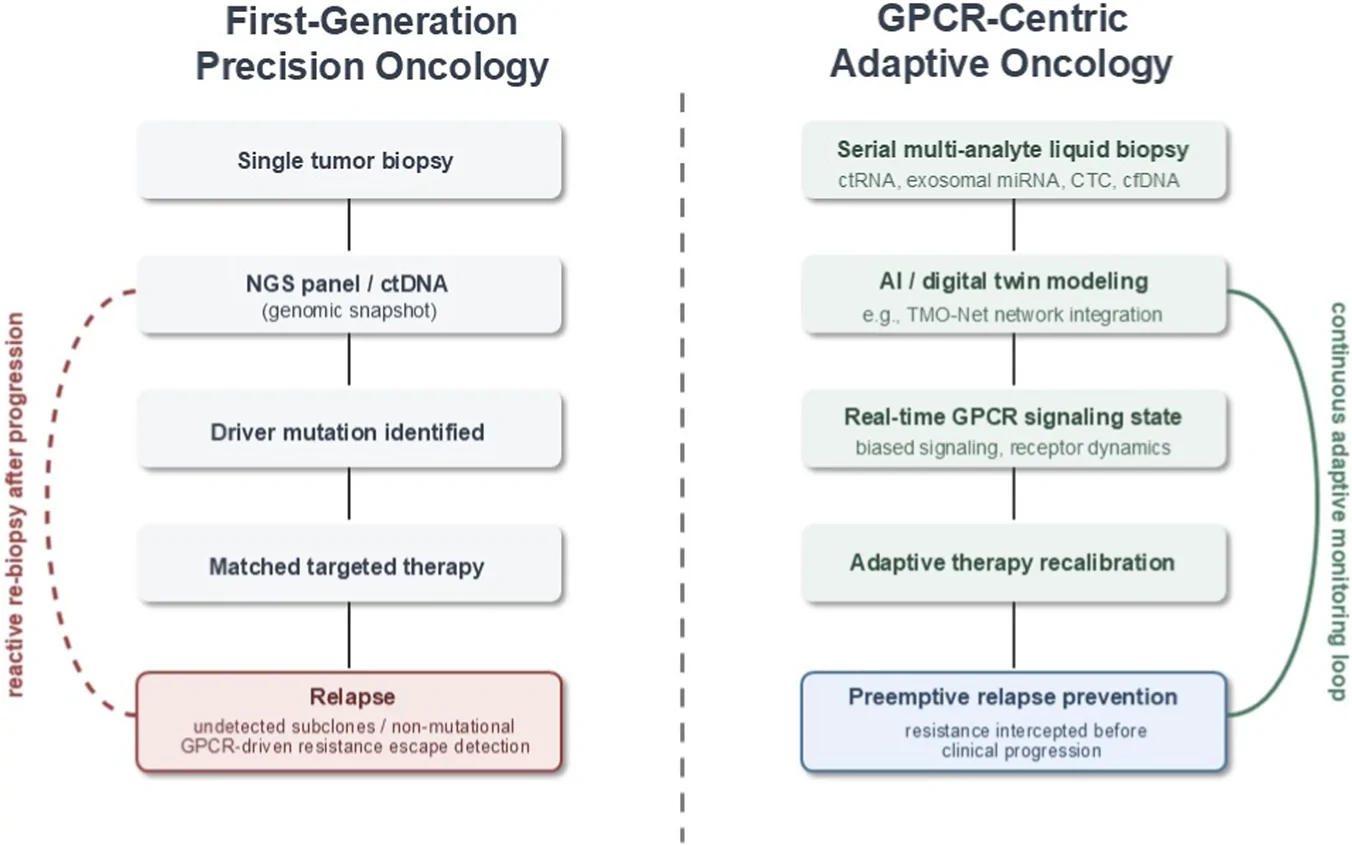
Transition from static, mutation-centric precision oncology to a dynamic, GPCR-informed adaptive framework. Comparison of first-generation and next-generation precision oncology workflows. Left panel: the conventional pipeline relies on a single tumor biopsy subjected to NGS/ctDNA panel sequencing to identify an actionable driver mutation, which is matched to a targeted therapy. Because this approach captures only a static genomic snapshot, it fails to detect non-mutational resistance mechanisms, including GPCR-driven transcriptional, epigenetic, and microenvironmental adaptations, resulting in relapse and reactive re-biopsy only after clinical progression. Right panel: the proposed GPCR-centric framework substitutes serial, multi-analyte liquid biopsy (ctRNA, exosomal miRNA, CTCs, cfDNA) for single-timepoint sampling. These longitudinal data feed AI-driven network models and digital twin platforms that resolve real-time GPCR signaling states, enabling adaptive therapy recalibration before resistance manifests clinically, within a continuous monitoring loop rather than a linear, one-shot pipeline.

This review was conducted as a narrative rather than systematic synthesis, reflecting the breadth of biological, technological, and clinical domains it spans. Relevant literature was identified through structured searches of PubMed/MEDLINE, Scopus, and Web of Science using combinations the terms including “GPCR” or “G protein-coupled receptor” with “precision oncology,” “liquid biopsy,” “ctRNA,” “digital twin,” “PROTAC,” and “biased agonism,” together with individual receptor or cancer-type identifiers (e.g., “CXCR4,” “FZD7,” “non-small cell lung cancer”). Searches were weighted toward publications from 2018 through 2026, with earlier foundational studies retained where mechanistically necessary. Candidate sources were prioritized for peer-reviewed status and direct relevance to GPCR biology in oncology, multi-omics integration, or liquid biopsy methodology; for clinical claims, priority was given to sources reporting a registered trial identifier or peer-reviewed clinical outcome data. Reviews were included selectively for context and synthesis rather than as substitutes for primary data. As a narrative rather than systematic review, this approach does not apply formal inclusion/exclusion screening (e.g., PRISMA) and cannot fully exclude selection bias favoring published positive findings.

It is important to situate this review relative to prior work addressing individual components of this landscape. The concept of an “Onco-GPCRome” of oncogenic receptor-driven networks has previously been articulated (Wu et al., 2019), as has the use of GPCR-informed digital twins as a standalone computational modeling tool (Boeringer et al., 2025), and the broader shift toward expression-based liquid biopsy within genomically framed precision oncology (Gulati et al., 2026). Each represents an important but partial view of this space. The distinguishing contribution of the present review is not any one of these observations individually, but their explicit synthesis into a single, iterative clinical framework: expression-based, multi-analyte liquid biopsy monitoring of GPCR signaling states, integrated through AI-driven network models and patient-specific digital twins, generating biomarker-triggered adaptive therapy decisions before resistance manifests clinically. To our knowledge, no prior review connects GPCR pharmacological plasticity, multi-analyte liquid biopsy, and digital-twin-guided adaptive trial design within one closed monitoring-and-recalibration loop.

Given the heterogeneous maturity of the technologies and modalities discussed throughout this review, we adopt a four-tier evidence classification to aid interpretation: (i) Clinical, referring to findings from completed or ongoing human trials or approved clinical use; (ii) Preclinical (*in vivo*), referring to findings validated in animal or patient-derived xenograft/organoid models; (iii) Preclinical (*in cellula*)/Computational, referring to cell-based, structural, or purely computational/predictive findings without *in vivo* validation; and (iv) Theoretical/conceptual, referring to frameworks proposed as a future direction rather than a demonstrated capability. This classification is applied consistently throughout and summarized for the principal technologies discussed in Supplementary Table S1. Notably, GPCR digital twins and AI-driven network models such as TMO-Net currently occupy the computational/predictive tier (Wang et al., 2024; Boeringer et al., 2025), and the closed-loop AI-integrated monitoring platform described in Section 6.3 is explicitly a theoretical/conceptual proposal rather than an existing clinical system.

## 2 Integrative genomics tools enabling GPCR-Centric precision oncology

### 2.1 Multi-omics profiling of GPCR networks

The advent of integrative multi-omics approaches has fundamentally expanded the resolution with which GPCR biology can be interrogated in cancer (Orsini et al., 2023). Bulk RNA sequencing, single-cell RNA sequencing (scRNA-seq), assay for transposase-accessible chromatin sequencing (ATAC-seq), and mass spectrometry-based proteomics together enable the comprehensive characterization of GPCR expression heterogeneity, epigenetic accessibility, and protein-level signaling states across diverse tumor types (Li et al., 2023). Critically, proteogenomic analyses have revealed that GPCR mutations do not reliably predict expression dysregulation, with a substantial proportion of GPCR upregulation in cancer occurring through non-mutational mechanisms such as promoter demethylation, enhancer rewiring, and post-transcriptional regulation (Xie et al., 2024; Arora et al., 2024). This decoupling of genomics from proteomics underscores the necessity of multi-modal profiling to fully capture the pathological nuances of oncogenic GPCR activation.

Pan-cancer multi-omics studies have identified GPCR hubs with prognostic and therapeutic relevance across tumor types. For example, in prostate cancer, integration of genomic, transcriptomic, and proteomic data has mapped the landscape of GPCR dysregulation throughout disease progression, identifying key receptors involved in androgen-independent adaptation and castration-resistant signaling (Li et al., 2022). In melanoma, combinatorial analysis of GPCR expression profiles and TME (tumor microenvironment) cellular composition has yielded actionable classifiers predictive of immunotherapy response and overall survival, highlighting the bidirectional crosstalk between GPCR signaling and immune cell recruitment (Shen et al., 2023). In lung adenocarcinoma, machine learning integration of multi-omics GPCR features has identified receptor combinations with superior predictive value over individual markers (Xie et al., 2024). These studies collectively reinforce the concept that GPCR networks function as dynamic hubs integrating diverse oncogenic inputs, and that their full characterization requires simultaneous profiling across genomic, transcriptomic, proteomic, and metabolomic dimensions (Maudsley et al., 2018; Maudsley et al., 2022; Leysen et al., 2018).

Beyond individual cancer types, multi-omics analyses have illuminated oncocrine networks in which GPCRs coordinate autocrine and paracrine signaling loops within the TME. Ligand gradients of chemokines, prostaglandins, lysophospholipids, and metabolites activate specific GPCR subtypes to orchestrate tumor angiogenesis, stromal remodeling, and immune exclusion. These non-cell-autonomous mechanisms represent an underappreciated dimension of oncogenic signaling that static genomic profiling is ill-equipped to resolve (Wu et al., 2019; Zhu et al., 2026; Arora et al., 2024).

### 2.2 NGS and single-cell technologies

High-resolution NGS has catalogued GPCR-linked somatic variants, copy number variations (CNVs), and fusion events at scale. A systematic pan-cancer analysis of TCGA data across 20 solid tumor categories (45 histological subtypes; n = 6,224 tumors for differential expression, n = 5,103 for mutation, and n = 7,545 for copy-number analyses) found that most tumor types differentially express more than 50 GPCRs relative to normal tissue, with differential expression showing greater prognostic relevance for survival than either mutation or copy-number variation (Sriram et al., 2019). In pancreatic adenocarcinoma specifically, GPRC5A was the most highly expressed GPCR, showing a 50-fold increase over normal pancreas, with 95% of tumor samples exhibiting at least an 8-fold increase in median expression (Sriram et al., 2019). These catalogues provide a genomic foundation for prioritizing GPCR targets for functional characterization and therapeutic development.

Single-cell multi-omics technologies have added a crucial layer of spatial and cellular resolution to GPCR biology. By enabling simultaneous profiling of transcriptomes, chromatin accessibility, and surface proteomes at single-cell resolution, these platforms reveal the heterogeneous GPCR expression landscapes within tumors, identifying rare subpopulations with distinct signaling states that may harbor pre-existing resistance or stem-like properties (Roy et al., 2026; Le et al., 2025; Huang et al., 2025). In glioblastoma, single-cell multi-omics analyses elucidated the role of GPR56 in mediating mesenchymal transition, a key resistance mechanism, demonstrating subpopulation-specific GPCR signaling that bulk profiling would have obscured (Ganesh et al., 2022). This functional data granularity is essential for designing *de novo* therapeutic strategies that preemptively target rare resistant clones before they achieve clonal dominance under treatment pressure.

Spatial transcriptomics technologies such as 10x Visium and MERFISH, further complement single-cell analyses by preserving tissue architecture, enabling the mapping of GPCR signaling gradients relative to the TME, vasculature, and immune infiltrate. Such spatial information is invaluable for understanding how ligand gradients drive GPCR activation in specific tumor niches, including hypoxic cores and invasive fronts (Liu et al., 2026; Cilento et al., 2024).

Beyond spatial transcriptomics, spatial proteomic platforms, including imaging mass cytometry and multiplexed immunofluorescence panels, extend this resolution to the protein level, enabling direct visualization of GPCR surface expression and its co-localization with immune and stromal populations within intact tissue architecture (Cilento et al., 2024). Multiplex imaging approaches of this kind are particularly well suited to resolving the intratumoral heterogeneity introduced in Section 1.2, in which genetically and epigenetically distinct subclones coexist within a single tumor mass (Zhu et al., 2021; Mubin et al., 2026); by preserving spatial context, they can localize rare GPCR-expressing subpopulations, such as the GPR56-driven mesenchymal subpopulations described in glioblastoma (Ganesh et al., 2022; Roy et al., 2026), to specific niches (e.g., the invasive tumor margin or hypoxic core) that bulk or dissociated single-cell approaches cannot resolve.

### 2.3 AI and network biology integration

The complexity of GPCR signaling networks, characterized by receptor promiscuity, signaling bias, context-dependent activation, and extensive crosstalk with other oncogenic pathways, renders them particularly amenable to AI-driven analytical frameworks (Craig et al., 2026; Junyent et al., 2025; Jobe and Vijayan, 2024; Wang et al., 2025). Multi-omics network integration models such as TMO-Net leverage graph neural networks to predict GPCR synthetic lethalities, identify resistance nodes, and prioritize combination therapy strategies from high-dimensional data (Liu et al., 2025). These tools move beyond correlative analyses to generate mechanistic hypotheses about GPCR network vulnerabilities in specific tumor contexts.

Chemokine-GPCR interaction networks exemplify the analytical power of AI in this domain. A recent comprehensive mapping of the human chemokine-GPCR interactome using deep mutational scanning and structural modeling revealed the molecular determinants of selectivity and promiscuity in chemokine receptor activation, identifying previously unappreciated receptor-ligand pairs with functional relevance to cancer immunology (Kleist et al., 2025). Such systematic interaction maps, integrated with patient-specific transcriptomic data, enable the prediction of dominant chemokine signaling axes in individual tumors, informing patient-stratified immunotherapy combination strategies.

Beyond network modeling, AI approaches are accelerating GPCR-targeted antibody development for CAR-T cell immunotherapy, identifying surface-expressed GPCRs as novel CAR-T targets, and optimizing antibody specificity against conformationally diverse receptor states (Liu et al., 2025). AI-driven structure-based drug design, powered by cryo-EM structural data and AlphaFold-derived models, is similarly transforming the identification of allosteric binding sites and biased agonist scaffolds for previously undruggable GPCRs (Zhang et al., 2024; Rubach et al., 2025; Liu X. et al., 2026).

### 2.4 Toward an integrated computational workflow

Sections 2.1–2.3 describe genomic, transcriptomic, proteomic, single-cell, and spatial technologies for characterizing GPCR biology largely as parallel analytical streams. Translating these into a single, clinically actionable model requires an explicit computational integration workflow (Supplementary Figure S1). Baseline tumor characterization (bulk RNA sequencing/whole-exome sequencing and single-cell RNA-seq/ATAC-seq) establishes the GPCR expression, mutational, and subclonal landscape (Sriram et al., 2019; Huang et al., 2025); spatial transcriptomic and proteomic data contextualize this landscape within the tumor microenvironment (Cilento et al., 2024); and serial liquid biopsy analytes, ctRNA, exosomal cargo, and circulating tumor cells, provide the longitudinal input layer capturing signaling dynamics over the course of treatment (Misawa et al., 2020; Tang et al., 2024). These heterogeneous streams are computationally harmonized, through batch correction and cross-platform feature selection, before being modeled by network-integration tools such as TMO-Net (Wang et al., 2024), which predicts candidate resistance nodes and combination targets. Model outputs are, where possible, cross-validated against patient-derived organoid drug-response data (Ovchinnikova et al., 2024) prior to informing a digital-twin-guided treatment recommendation (Boeringer et al., 2025) and, ultimately, a biomarker-triggered adaptive or basket-trial therapy switch (Ha et al., 2024). Serial re-sampling following any such switch closes the loop, feeding updated liquid biopsy data back into the model for the next iteration. We emphasize that while each individual step in this workflow has independent methodological support, the fully integrated pipeline as depicted has not yet been demonstrated end-to-end in a prospective clinical study; its presentation here is intended as a conceptual roadmap (Supplementary Table S1).

## 3 Liquid biopsy evolution for GPCR monitoring

### 3.1 Beyond ctDNA: expression-based liquid biopsy modalities

While circulating tumor DNA (ctDNA) established the foundation of liquid biopsy-guided oncology, its inherent limitation to genetic-level information leaves the investigation of functional signaling landscapes, including GPCR expression dynamics, relatively inaccessible to this technology. The next-generation, however, of liquid biopsy platforms integrates multiple analyte classes to capture a holistic view of tumor biology in real time. Circulating tumor RNA (ctRNA), exosomal microRNA, long non-coding RNAs (lncRNAs), and extracellular vesicle-associated proteins collectively provide expression-level information that reflects the transcriptional and signaling states of tumors at the time of sampling (Misawa et al., 2020; Tang et al., 2024).

ctRNA profiling is particularly productive for monitoring GPCR expression changes that are invisible to DNA-based approaches. Therapy-induced transcriptional upregulation of chemokine receptors, epigenetic reactivation of silenced GPCRs, and ligand-dependent receptor recycling can all be captured through ctRNA analysis, enabling real-time tracking of non-mutational resistance mechanisms (Gulati et al., 2026). Furthermore, GPCR genes including PTGDR1, PTGDR2, and PTGIR have been identified as epigenetically regulated biomarkers detectable in liquid biopsies of HPV-associated oropharyngeal cancer, illustrating the potential of methylation-based GPCR biomarker panels complementing expression-level analysis (Misawa et al., 2020; Chen et al., 2026).

### 3.2 Multi-analyte integration and comprehensive GPCR profiling

The most informative liquid biopsy strategies combine circulating tumor cells (CTCs), cell-free DNA (cfDNA), ctRNA, and exosomal cargo in integrated multi-analyte panels that provide complementary information about tumor genetics, epigenetics, transcriptomics, and proteomics simultaneously (Tang et al., 2024). This multi-analyte approach substantially enhances sensitivity for detecting early adaptive signals, particularly in the context of minimal residual disease and emergent resistance, where single-analyte platforms may miss subclonal alterations below their limit of detection.

Exosomes merit particular attention as vehicles for GPCR-associated signaling information. Tumor-derived exosomes carry receptor proteins, signaling lipids, and non-coding RNAs that reflect the signaling state of their cells of origin, and their cargo composition changes in response to therapy-induced stress (Isola and Chen, 2016). Serial profiling of exosomal GPCR content therefore offers a non-invasive window into the real-time rewiring of tumor signaling networks (Ofori et al., 2020). Advanced molecular and functional profiling platforms that combine targeted DNA sequencing with exosomal RNA and protein analysis are enabling serial assessment of GPCR pathway activity to inform adaptive treatment switches, representing a significant advance over static genomic profiling (Tang et al., 2024) (Figure 2).

**FIGURE 2:**
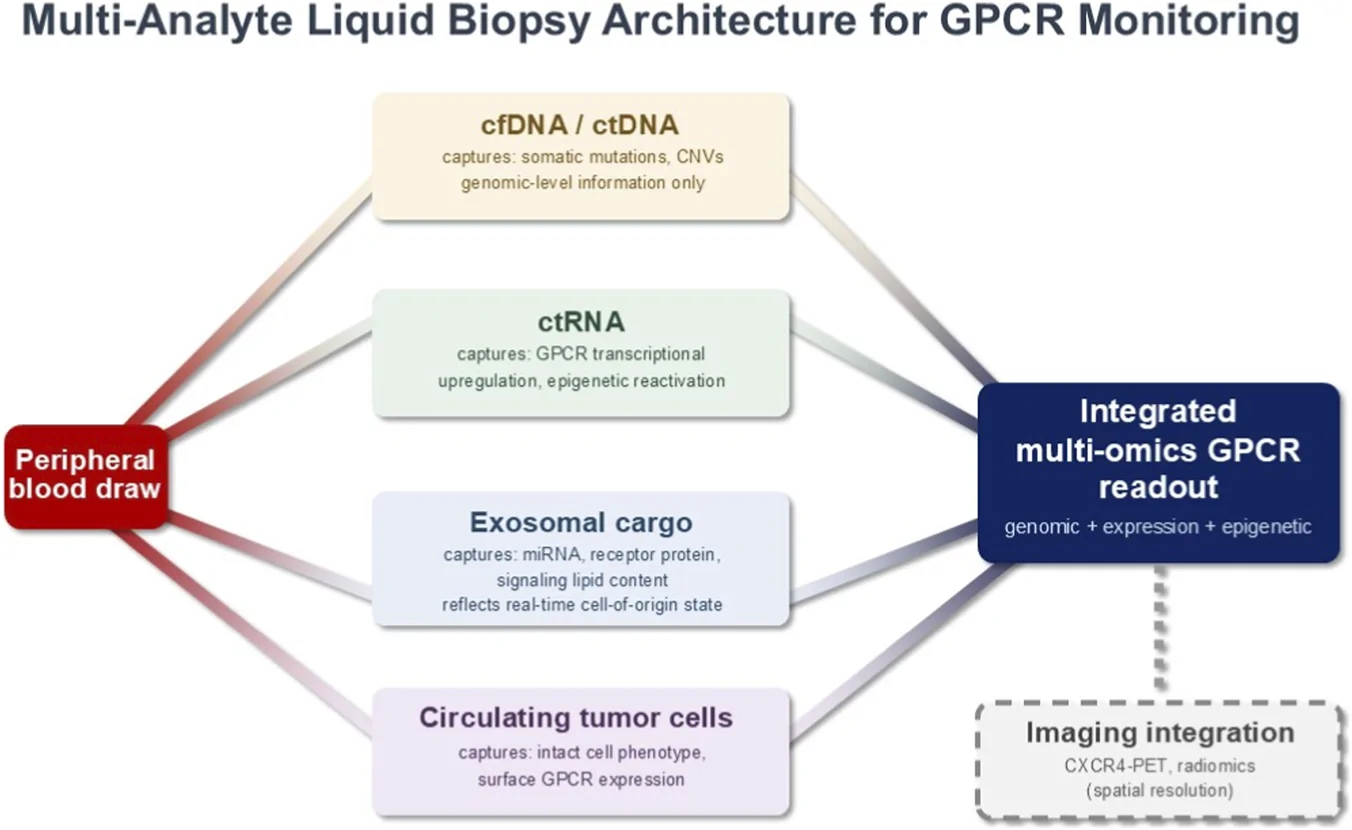
Complementary liquid biopsy analyte streams converge to provide a functional, non-invasive readout of GPCR signaling state. Schematic of peripheral blood-derived analyte classes and the distinct layers of biological information each captures. cfDNA/ctDNA provides genomic-level information (somatic mutations, copy number variants) but cannot detect non-mutational GPCR dysregulation. ctRNA captures transcriptional upregulation and epigenetic reactivation of GPCR genes. Exosomal cargo (miRNA, receptor protein, signaling lipids) reflects the real-time signaling state of cells of origin. Circulating tumor cells (CTCs) preserve intact cellular phenotype and surface GPCR expression. These streams converge into an integrated multi-omics GPCR readout, which can be further enriched by spatially resolved imaging modalities (e.g., CXCR4-targeted PET, radiomics) shown in the dashed inset.

### 3.3 Imaging-integrated liquid biopsy platforms

Emerging investigational initiatives can integrate liquid biopsy GPCR monitoring with advanced precision imaging modalities, including radiomics, molecular imaging, and GPCR-targeted PET tracers, to provide spatially resolved information about receptor expression *in vivo* (Hricak et al., 2025). CXCR4-targeted PET imaging, for example, enables whole-body mapping of receptor expression in hematologic malignancies and solid tumors, providing spatial context that complements the aggregate signal of blood-based liquid biopsies. Integration of these imaging-liquid biopsy platforms with AI-driven interpretation frameworks represents a powerful approach to achieving comprehensive, spatiotemporally resolved GPCR monitoring in clinical practice (Cheng et al., 2024; Yang et al., 2026).

## 4 GPCRs in tumor evolution and adaptive therapy

The oncogenic contributions of GPCRs described in this section are not homogeneous across the receptor superfamily, and it is useful to organize them by receptor family and canonical signaling logic before considering individual mechanisms. Class A chemokine receptors, exemplified by CXCR4, are most consistently implicated in metastatic homing, bone marrow niche retention, and immune evasion, acting through the classical G protein/β-arrestin signaling repertoire of this receptor class (Furusato et al., 2010; Hamshaw et al., 2023; Kleist et al., 2025). Class F Frizzled receptors, exemplified by FZD7, function as Wnt co-receptors and are most consistently implicated in epithelial-to-mesenchymal transition (EMT), cancer stem cell maintenance, and therapy resistance (Liu et al., 2019; Dreyer et al., 2023). Adhesion GPCRs, exemplified by GPR56, similarly contribute to mesenchymal transition programs, particularly in glioblastoma (Ganesh et al., 2022). Proton-sensing Class A receptors (GPR65, GPR4, GPR68) in contrast couple extracellular acidification, a hallmark of the glycolytic tumor microenvironment, to pro-survival and pro-migratory transcriptional programs (Ahn and Kaipparettu, 2025; Cornell et al., 2024). Finally, aminergic Class A GPCRs, while less directly oncogenic themselves, provide the best-characterized structural and pharmacological template for β-arrestin-biased ligand design, informing biased-ligand strategies now being extended to oncology-relevant receptors (McCorvy et al., 2018). This receptor-family organization, summarized in Supplementary Table S2, underscores that “the GPCR” is not a single therapeutic entity but a superfamily whose oncogenic relevance and druggability vary meaningfully by class.

### 4.1 Resistance mechanisms mediated by GPCR signaling

GPCRs constitute convergence nodes for multiple non-genetic resistance mechanisms that emerge under therapeutic pressure. Therapy-induced metabolic stress, hypoxia, acidosis, and immune editing collectively reshape the ligand microenvironment in ways that selectively activate specific GPCR subtypes to promote tumor cell survival, invasion, and immune evasion (Craig et al., 2026; Cornell et al., 2024; Kumari et al., 2021). Proton-sensing GPCRs, including GPR65, GPR4, and GPR68, are upregulated in acidic TMEs arising from tumor glycolytic metabolism, where they transduce extracellular pH signals into pro-survival and pro-migratory intracellular programs (Ahn and Kaipparettu, 2025; Cornell et al., 2024; Guillemette et al., 2026). This metabolic adaptation represents a purely non-genetic resistance axis invisible to DNA sequencing.

The Frizzled receptor FZD7, a GPCR in the Wnt signaling pathway, exemplifies the clinical significance of GPCR-driven resistance in glioblastoma. FZD7 overexpression drives mesenchymal transition and enhances resistance to temozolomide and radiation and has been validated as a therapeutic target amenable to small molecule inhibition (Liu et al., 2019; Azam et al., 2020; Dreyer et al., 2023); Cycloartobiloxanthone has been identified as a promising FZD7 inhibitor across multiple patient cohorts (Sun et al., 2025). Similarly, GPCR-mediated transactivation of receptor tyrosine kinases (RTKs) via c-Src, in which GPCR activation triggers RTK phosphorylation in the absence of growth factor ligands, represents a well-characterized bypass mechanism enabling continued proliferative signaling even under RTK-targeted therapy (Almendro et al., 2010).

These GPCR-driven resistance and adaptation mechanisms do not operate in isolation from the oncogenic signaling pathways more traditionally targeted in precision oncology, and it is useful to make this crosstalk explicit (Supplementary Figure S2). Gβγ- and Gαq-coupled GPCR signaling engages the PI3K–AKT axis, linking receptor activation to the metabolic reprogramming discussed above (Ahn and Kaipparettu, 2025). Both G protein- and β-arrestin-biased GPCR signaling converge on the canonical RAS–ERK (MAPK) cascade (Eiger et al., 2022; McCorvy et al., 2018). FZD7, as a Wnt co-receptor, directly couples GPCR-family signaling to canonical Wnt/β-catenin activity and downstream EMT programs (Liu et al., 2019; Dreyer et al., 2023). GPCR signaling also activates the Hippo pathway effectors YAP/TAZ, a mechanism shown to drive resistance to both MEK inhibition and immunotherapy in uveal melanoma (Faião-Flores et al., 2019; Ren et al., 2026). Finally, GPCR-driven oncocrine signaling cross-regulates TGF-β-dependent EMT and stromal remodeling programs within the tumor microenvironment (Wu et al., 2019). Recognizing these intersections reframes GPCRs not as a parallel, independent oncogenic axis, but as a signaling layer mechanistically interwoven with the RTK, PI3K–AKT, MAPK, WNT, Hippo, and TGF-β pathways already central to precision oncology.

GPCRs also contribute to metabolic reprogramming in cancer through the modulation of lipid metabolism, glutamine utilization, and mitochondrial biogenesis, connecting receptor signaling to bioenergetic adaptations that sustain tumor growth under nutrient-deprived conditions (Ahn and Kaipparettu, 2025; Kumari et al., 2021; Guillemette et al., 2026). These metabolic connections underscore the broad role of GPCR networks as integrators of environmental stress signals into adaptive cellular responses (Boeringer et al., 2026; Maudsley et al., 2022a).

### 4.2 Serial monitoring of GPCR adaptations in metastatic disease

The concept of serial tumor monitoring, analogous to the established paradigm of tracking EGFR resistance mutations in lung cancer, is being extended to GPCR expression dynamics in metastatic settings (Maheswaran et al., 2008; Le et al., 2025; Khan et al., 2020). In metastatic breast cancer, serial ctDNA and transcriptomic profiling have demonstrated dynamic CXCR4 upregulation during disease progression, correlating with metastatic dissemination and immune exclusion in the TME (Hamshaw et al., 2023). Mechanistically, CXCR4 activation by its ligand CXCL12 promotes directional tumor cell migration toward bone marrow and lymph node niches, and CXCR4 antagonists, including AMD3100 and more potent competitive inhibitors, have demonstrated preclinical efficacy in disrupting metastatic homing (Hamshaw et al., 2023). Novel molecular trap approaches using water-solubilized CXCR4QTY-Fc fusion proteins have shown promise in inhibiting metastatic dissemination by sequestering CXCL12 (Sun C. et al., 2025).

These serial monitoring strategies depend critically on the sensitivity and specificity of the liquid biopsy platforms employed. In genitourinary cancers, where precision medicine has historically faced significant challenges related to tumor heterogeneity and limited actionable genomic alterations, serial expression-based liquid biopsy offers a promising avenue for capturing GPCR-driven adaptive signaling that would otherwise precipitate treatment failure (Fontes, 2024).

### 4.3 GPCR-specific examples across tumor types

GPRC5A (Retinoic Acid-Induced Protein 3, RAI3) has emerged as a compelling pan-cancer GPCR with metastatic relevance. In NSCLC, GPRC5A overexpression correlates with poor survival and is associated with enhanced immune cell exclusion, immune evasion, and activation of oncogenic signaling cascades; liquid biopsy tracking of GPRC5A expression levels has been proposed as a real-time prognostic biomarker (Lin et al., 2024). In a cohort of NSCLC patients from the Fudan University Shanghai Cancer Center, low GPRC5A expression was specifically associated with earlier pathological stage and with increased infiltration of CD8^+^ T cells, activated CD4^+^ T cells, and M1 macrophages, consistent with a role for GPRC5A upregulation in immune cell exclusion (Lin et al., 2024). In esophageal squamous cell carcinoma, GPRC5A facilitates early pulmonary colonization by promoting the survival and proliferation of circulating tumor cells in the pulmonary microenvironment, identifying it as a potential target for preventing metastatic seeding (Zhou et al., 2024). The mechanistic parallels between GPRC5A function in these histologically distinct cancers exemplify the pan-cancer oncogenic potential of certain GPCRs and support tumor-agnostic therapeutic strategies targeting conserved GPCR signaling axes (Figure 3).

**FIGURE 3:**
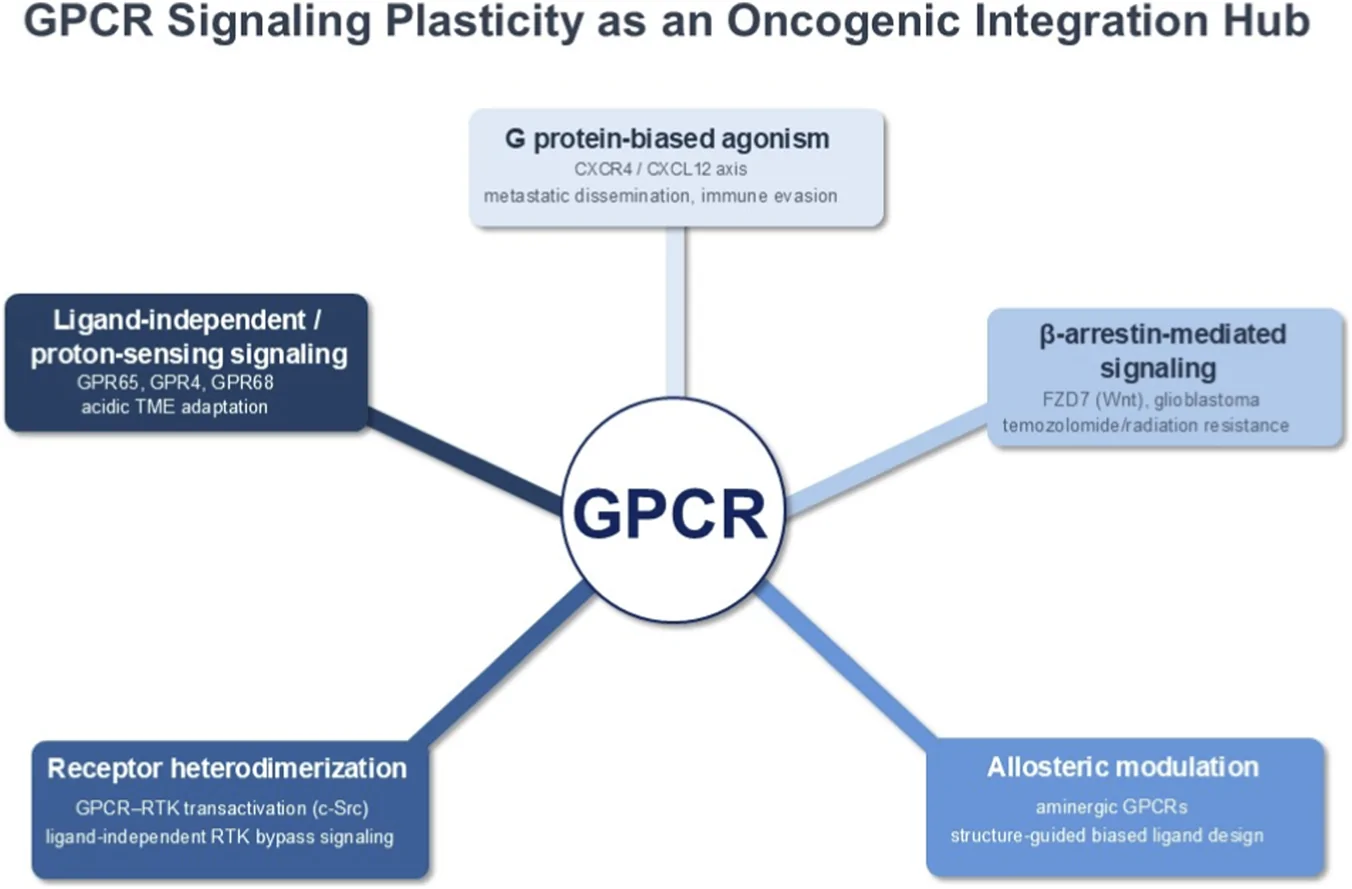
Five modes of GPCR pharmacological plasticity underlying oncogenic signaling. Schematic of the central GPCR signaling node and its five principal modes of pharmacological engagement relevant to cancer biology: G protein-biased agonism (exemplified by the CXCR4/CXCL12 axis in metastatic dissemination and immune evasion), β-arrestin-mediated signaling (FZD7-driven Wnt pathway activation and mesenchymal transition in glioblastoma, conferring temozolomide/radiation resistance), allosteric modulation (structure-guided biased ligand design at aminergic GPCRs), receptor heterodimerization (GPCR-RTK transactivation via c-Src enabling ligand-independent bypass signaling), and ligand-independent/proton-sensing signaling (GPR65, GPR4, and GPR68 sensing of the acidic tumor microenvironment). Together these mechanisms illustrate why GPCRs function as multifunctional integration hubs rather than simple on/off signaling switches.

Frizzled receptors beyond FZD7 represent a broader class of GPCR-Wnt co-receptors with roles in cancer stem cell maintenance, epithelial-to-mesenchymal transition (EMT), and therapy resistance across multiple tumor lineages. Their druggability is being actively explored through small molecule allosteric modulators, antibody-drug conjugates, and PROTAC-mediated degradation strategies.

### 4.4 Synergies between GPCR inhibitors and immunotherapy

The immunomodulatory functions of GPCRs within the TME position them as rational combination partners for immune checkpoint inhibitors (ICIs) (Stagg and Gutkind, 2025; Dickinson et al., 2024). GPCR signaling in tumor-infiltrating lymphocytes, myeloid cells, and natural killer cells shapes their activation state, migratory capacity, and effector function (Ren et al., 2026). CXCR4 antagonism, for example, has been shown to mobilize immunosuppressive myeloid populations from bone marrow niches and reprogram the immunosuppressive TME toward an immune-permissive state, enhancing the efficacy of PD-1/PD-L1 blockade in preclinical models (Liu et al., 2025). Similarly, GPCRs coupled to the adenylyl cyclase-cAMP axis modulate T cell receptor signaling and can be targeted to overcome T cell exhaustion in the immunosuppressive TME (Wu et al., 2023; Yu et al., 2026).

An instructive example of multi-target GPCR-immunotherapy synergy comes from uveal melanoma, where HDAC inhibition was shown to enhance MEK inhibitor efficacy specifically by suppressing GPCR-mediated YAP/TAZ activation, a transcriptional co-activator pathway that drives resistance to both targeted therapy and immunotherapy (Faião-Flores et al., 2019). This mechanistic insight illustrates how GPCR signaling can serve as a master regulator of transcriptional programs that must be co-targeted to achieve durable antitumor responses. Biased GPCR agonists that selectively engage anti-tumorigenic signaling arms while avoiding pro-tumorigenic pathways represent a sophisticated strategy for exploiting these immunomodulatory functions in combination immunotherapy regimens (Ren et al., 2026; Zhai et al., 2026).

### 4.5 Limitations, controversies, and negative or mixed findings

The preceding sections emphasize the therapeutic promise of GPCR-centric oncology, but a balanced account requires acknowledging substantial unresolved tensions in the field. First, despite the ∼800-member size of the GPCR superfamily and its status as the most heavily drugged gene family in other therapeutic areas, oncology-specific GPCR-targeted approvals remain few, and translation of GPCR pharmacology from cardiovascular and CNS indications into durable oncology benefit has historically proven difficult. Second, receptor promiscuity and pathway redundancy, exemplified by the extensive cross-reactivity mapped across the human chemokine-GPCR interactome (Kleist et al., 2025), can blunt the efficacy of single-receptor blockade and complicate attribution of resistance to any one GPCR. Third, chronic engagement of individual GPCR targets carries on-target toxicity risk: CXCR4 antagonism mobilizes hematopoietic stem and progenitor cells as an intended pharmacodynamic effect in transplantation but represents an unwanted systemic effect during prolonged oncology dosing, and the anti-Frizzled antibody vantictumab, despite an encouraging 31.3% objective response rate in combination with paclitaxel in HER2-negative breast cancer, saw its clinical development curtailed after six of 48 patients developed bone fractures attributable to on-target inhibition of Wnt signaling in bone (Diamond et al., 2020). Fourth, reported benefits are often modest or cohort-specific: the COMBAT trial combining the CXCR4 antagonist motixafortide with pembrolizumab and chemotherapy in metastatic pancreatic cancer reported a disease control rate of 34.5% in a chemotherapy-resistant cohort with a median overall survival of only 3.3 months in the intention-to-treat population, improving to 7.5 months when used in the second-line setting (Bockorny et al., 2020), underscoring that CXCR4 blockade, while biologically active, has not yet translated into transformative single-agent benefit. Fifth, the mechanistic link between GPCR signaling and Hippo/YAP-TAZ-driven resistance in uveal melanoma (Faião-Flores et al., 2019) rests on a single preclinical study rather than a validated combination regimen, and, more broadly, no prospective clinical trial has yet demonstrated that a liquid-biopsy-detected change in GPCR expression, used explicitly to trigger a treatment switch, improves patient outcomes; this central premise of the adaptive framework proposed in Section 6.3 therefore remains a hypothesis rather than an established practice. Collectively, these findings argue for cautious optimism: GPCR-centric precision oncology represents a biologically well-motivated and partially clinically validated direction, but one whose translational maturity varies considerably across receptors, modalities, and cancer types, and whose adoption should proceed alongside, rather than in place of, rigorous prospective validation.

## 5 Precision GPCR targeting: from discovery to clinic

### 5.1 Platforms for multidimensional GPCR drug discovery

The identification of precision GPCR therapeutics for oncology demands discovery platforms that can capture the full pharmacological complexity of receptor signaling, including biased agonism, allosteric modulation, and pathway-selective engagement (Eiger et al., 2022; Zhou et al., 2026; Jobe and Vijayan, 2024). High-throughput CRISPR-based platforms such as DCyFIR (Dynamic Cyan Inducible FIngeRprint) enable multiplexed GPCR profiling by simultaneously measuring receptor activation across multiple signaling readouts in a single assay, dramatically accelerating the identification of biased ligands with selective pathway engagement profiles (Kapolka et al., 2020). This multiplexed approach is particularly valuable for discovering compounds that selectively activate anti-tumor GPCR signaling arms (e.g., β-arrestin-mediated receptor internalization that attenuates oncogenic G protein signaling) while avoiding pro-tumorigenic effector pathways (Cahill et al., 2017; van Gastel et al., 2018).

Proteolysis-targeting chimera (PROTAC) technology has been applied to GPCRs, offering a fundamentally distinct mechanism of pharmacological intervention: rather than blocking receptor signaling, PROTACs recruit E3 ubiquitin ligases to induce targeted proteasomal degradation of overexpressed receptors (Saca et al., 2025). This approach is particularly relevant for GPCRs whose oncogenic functions involve scaffolding or protein-protein interaction roles not addressable by conventional orthosteric or allosteric ligands. GPCR-directed PROTACs have shown promising preclinical activity and represent a significant expansion of the druggable GPCR space in oncology (Saca et al., 2025; Faryal et al., 2026).

Cryo-electron microscopy (cryo-EM) has revolutionized structural biology of GPCRs by enabling atomic-resolution visualization of receptor complexes in multiple activation states, including G protein-coupled, arrestin-coupled, and ligand-free conformations. These structural insights, combined with AI-powered drug design tools, including generative molecular design and virtual screening against cryo-EM-derived binding pocket models, accelerating the identification of highly selective allosteric modulators for previously intractable GPCR targets (Shihoya et al., 2024; Singh et al., 2026).

### 5.2 Translational ecosystems and patient-specific models

The translation of GPCR discoveries from bench to bedside is increasingly supported by an ecosystem of academic-industry partnerships, biotech ventures, and translational platforms focused on age-related oncology indications. Digital twin technology represents one of the most promising translational tools in this landscape (Bouriga et al., 2025). GPCR digital twins, computational models integrating multi-omics patient data to simulate receptor network behavior, enable patient-specific prediction of drug responses, resistance trajectories, and optimal combination strategies (Boeringer et al., 2025). These models are trained on large patient cohort datasets and continuously updated with longitudinal liquid biopsy data, enabling genuine adaptive personalization of therapy over a patient’s treatment course.

Patient-derived organoids (PDOs) and co-culture systems incorporating immune cell components and stromal elements provide complementary *ex vivo* platforms for functional validation of GPCR-targeting strategies in patient-specific contexts. Integration of PDO drug response data with digital twin models enables the cross-validation of computational predictions with experimental evidence, enhancing the reliability of *in silico* therapy optimization (Ovchinnikova et al., 2024). AI-driven approaches to overcoming current limitations in drug response measurement, including spatiotemporal heterogeneity in organoid drug sensitivity, are further enhancing the predictive power of these platforms for clinical translation.

Biased ligands for aminergic GPCRs, whose structural basis has been illuminated by cryo-EM and computational modeling, exemplify the translational trajectory from structural insight to optimized clinical candidates (McCorvy et al., 2018). Structure-inspired design of β-arrestin-biased ligands for oncology-relevant aminergic receptors offers a route toward functional selectivity that maximizes anti-tumor efficacy while minimizing on-target toxicity, a critical consideration for chronic administration in cancer patients (McCorvy et al., 2018; Krumm and Roth, 2025).

### 5.3 RNA therapeutics targeting undruggable GPCRs

A significant proportion of oncologically relevant GPCRs lack suitable binding pockets for small molecule intervention, rendering them “undruggable” by conventional pharmacological approaches. RNA therapeutics, including antisense oligonucleotides (ASOs), siRNA, mRNA, and CRISPR-based transcriptional modulators, offer sequence-specific strategies for modulating GPCR expression regardless of protein structural tractability (Shah and Meric-Bernstam, 2025; Yuan et al., 2024). Tumor-agnostic RNA therapeutic approaches that target conserved GPCR signaling hubs across cancer types represent an emerging Frontier for expanding the actionable GPCR target space and complementing the limitations of small molecule and antibody-based modalities (Wu and Thawani, 2025; Santhanam et al., 2024).

## 6 Future directions for intelligent GPCR-Centric oncology

### 6.1 Technical and standardization challenges

Despite the transformative promise of GPCR-focused integrative oncology, significant technical challenges must be addressed before these approaches can achieve broad clinical implementation. Liquid biopsy sensitivity for GPCR RNA signals remains variable across platforms, with pre-analytical factors, including blood processing protocols, RNA preservation conditions, and sequencing depth, introducing substantial inter-laboratory variability (Rituraj et al., 2025; Maass et al., 2021; Strati et al., 2025). Standardization of multi-omics data processing pipelines, reference standards, and bioinformatic workflows is critical to ensure reproducibility and comparability of GPCR biomarker measurements across clinical sites (Van Hul and Cani, 2026).

The interpretive complexity of GPCR signaling data presents an additional challenge. Given the promiscuity of ligand-receptor interactions, the context-dependency of signaling bias, and the crosstalk between receptor families, computational frameworks for translating raw multi-omics GPCR data into actionable clinical insights require rigorous validation in prospective clinical datasets (Przydzial et al., 2013; Dankwah et al., 2022). Furthermore, prohibitive costs and limited accessibility in low-resource settings continue to restrict the implementation of advanced liquid biopsy and multi-omics platforms, necessitating the development of cost-effective, scalable assay formats (Rituraj et al., 2025).

A further challenge specific to GPCR monitoring is the comparatively low abundance of ctRNA relative to ctDNA, which limits sensitivity in low tumor-burden or early-stage settings and has no equivalent, in this domain, to the harmonized reference standards and proficiency-testing schemes now available for ctDNA (Maass et al., 2021; Strati et al., 2025). Exosome-based approaches introduce an additional source of inter-laboratory variability, since exosome isolation methodology, whether by ultracentrifugation, size-exclusion chromatography, or precipitation-based kits, is not yet standardized across the field and can materially affect the recovered GPCR-associated cargo (Ofori et al., 2020). Until such analytical standardization is achieved, comparisons of GPCR expression signals across studies and institutions should be interpreted cautiously.

### 6.2 Clinical trial design and adaptive oncology

The realization of GPCR-centric adaptive oncology requires clinical trial frameworks that can accommodate the dynamic, biomarker-guided treatment adjustments central to this paradigm. Adaptive trial designs incorporating prespecified interim analyses triggered by serial liquid biopsy GPCR biomarker thresholds enable mid-trial therapeutic recalibration, potentially improving response rates and reducing exposure to ineffective treatments (Ha et al., 2024; Guan et al., 2026; Zhou et al., 2025). A systematic analysis of Phase II clinical trials with biomarker-driven adaptive designs highlights the feasibility of this approach and identifies key design considerations, including pre-specification of biomarker thresholds, adaptive randomization rules, and statistical frameworks for multi-arm biomarker-stratified comparisons (Ha et al., 2024).

Tumor-agnostic trial designs that enroll patients based on GPCR expression or signaling profiles rather than tumor histology represent a particularly promising extension of the precision oncology paradigm, enabling the efficient evaluation of GPCR-targeted therapies across multiple cancer types in a single protocol (Shah and Meric-Bernstam, 2025; Fountzilas et al., 2024). Basket and umbrella trial architectures, already established for genomic precision oncology, provide adaptable templates for GPCR-expression-guided enrollment strategies (Duan et al., 2024; Chigozie and Esimone, 2026).

### 6.3 Vision for AI-integrated GPCR monitoring platforms

The long-term vision for GPCR-centric precision oncology is an AI-integrated clinical platform that continuously synthesizes multi-analyte liquid biopsy data, genomic, epigenomic, transcriptomic, and proteomic, with digital twin models of patient-specific GPCR signaling networks to generate real-time adaptive therapy recommendations. This platform would leverage predictive AI algorithms trained on large, annotated patient cohorts to anticipate resistance trajectories before they manifest as clinical progression, enabling preemptive therapeutic recalibration rather than reactive second-line switching (Tang et al., 2024; Boeringer et al., 2025; Li et al., 2026; Guan et al., 2026).

Integration with precision imaging modalities and electronic health record data would further enrich the contextual information available to these AI platforms, enabling holistic modeling of tumor-host interactions that account for comorbidities, pharmacokinetic variability, and immune context (Hricak et al., 2025). Federated learning approaches, which enable AI model training across distributed clinical datasets without sharing individual patient data, offer a privacy-preserving route to assembling the large, diverse training cohorts required to develop robust, generalizable GPCR predictive models. The convergence of these technological streams’ positions GPCR-centric integrative oncology as a foundational component of the next-generation of intelligent precision therapeutics (Rieke et al., 2020; Horst et al., 2025) (Figure 4).

**FIGURE 4:**
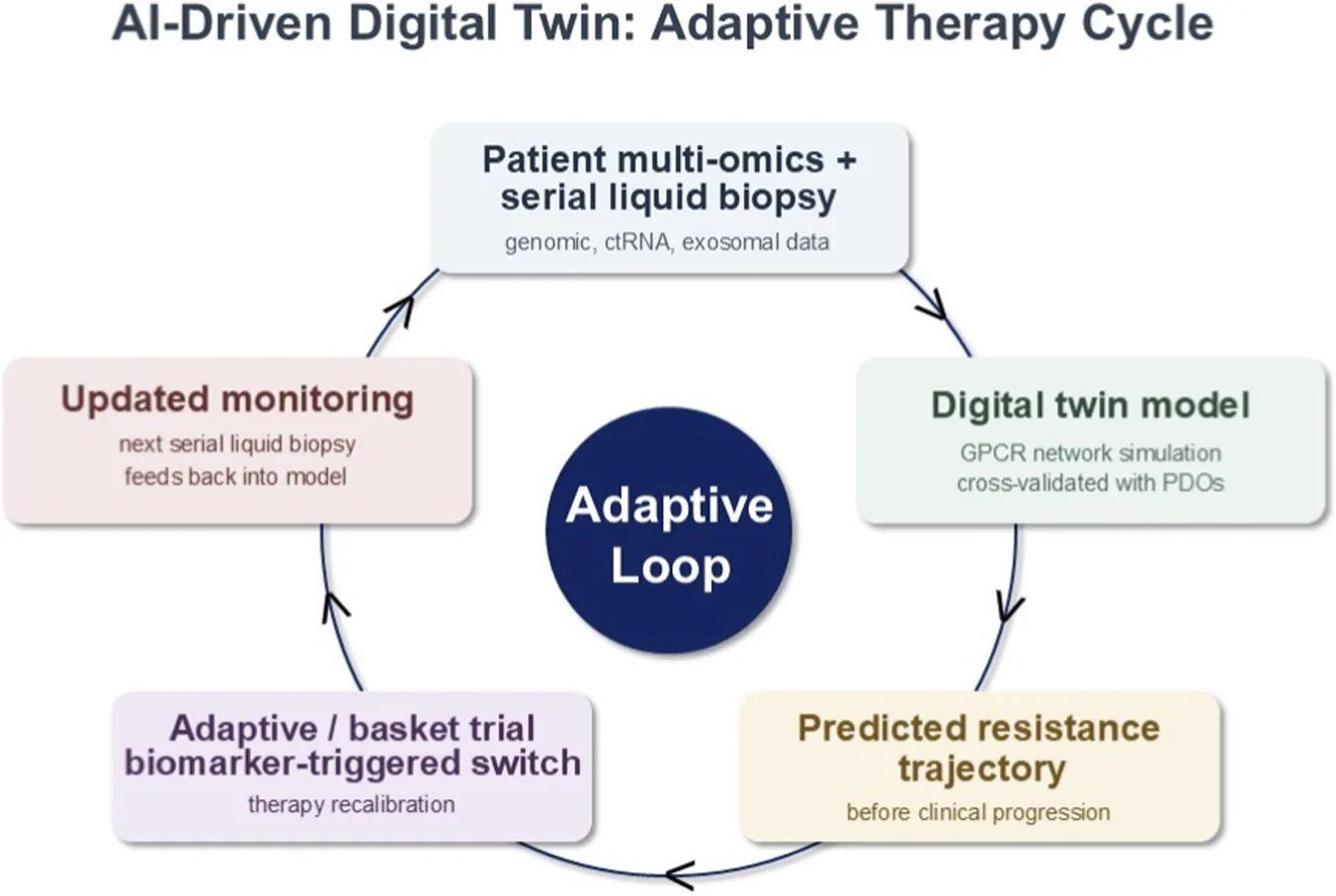
Closed-loop cycle linking longitudinal multi-omics data, digital twin modeling, and biomarker-triggered adaptive trial design. Conceptual workflow illustrates how patient-specific multi-omics and serial liquid biopsy data inform a computational digital twin of GPCR network behavior, cross-validated against patient-derived organoid (PDO) drug response data. The digital twin generates predictions of resistance trajectories in advance of clinical progression, which trigger biomarker-defined adaptive or basket trial therapy switches. Updated monitoring following the therapy change feeds the next round of liquid biopsy data back into the model, closing the loop and enabling continuous, iterative treatment optimization over the course of a patient’s care.

### 6.4 From framework to clinical practice

Realizing the framework described above will require a concrete implementation pathway rather than technological capability alone. Patient selection would reasonably begin with tumor types already showing documented GPCR dysregulation, including NSCLC, melanoma, prostate, pancreatic, and glioblastoma, where the receptor-level evidence summarized in Supplementary Table S2 is strongest. Before clinical deployment, any GPCR-focused liquid biopsy assay would need to progress through the same validation tiers established for existing ctDNA assays: analytical validity (sensitivity, specificity, and reproducibility across sites and instruments), clinical validity (a defined association with outcome in a specified population), and clinical utility (demonstrated improvement in patient management or outcome in a prospective trial). Depending on its intended use, such an assay would likely be developed either as a laboratory-developed test under CLIA/CAP oversight or, for broader adoption, as an FDA-regulated companion diagnostic; the latter pathway would require the kind of prospective, biomarker-triggered adaptive trial design discussed in Section 6.2. We are not aware of a published health-economic analysis of GPCR-guided liquid biopsy, and formal cost-effectiveness modeling, benchmarked against the added cost of multi-analyte sampling and computational infrastructure relative to standard genomic panels, is itself a necessary area of future work rather than an established finding. This implementation gap is compounded by disparities in access. As illustrated by the experience of NSCLC precision oncology within Brazil’s public healthcare system (Schneider et al., 2025), many patients globally cannot access even first-generation genomic precision oncology, let alone multi-analyte liquid biopsy and AI/digital-twin infrastructure layered on top of it. The sequencing capacity, computational infrastructure, and data-storage resources required for the framework proposed in Section 6.3 are unevenly distributed worldwide, and without deliberate mitigation, GPCR-guided precision oncology risks widening, rather than narrowing, existing disparities in precision oncology access (Rituraj et al., 2025). Federated learning approaches, which enable model training across distributed clinical datasets without centralizing patient-level data (Rieke et al., 2020; Horst et al., 2025), and the development of lower-cost, scalable assay formats represent partial mitigation strategies, though neither has yet been demonstrated to close this gap in practice.

## 7 Conclusion

Intelligent precision oncology, powered by integrative genomics, expression-based liquid biopsy monitoring, and GPCR-focused adaptive therapeutics, represents a paradigm-shifting evolution in cancer medicine. The recognition of GPCRs as dynamic oncogenic hubs, integrating extracellular cues from the tumor microenvironment with intracellular transcriptional programs via pharmacologically complex signaling networks, elevates them from underappreciated background players to central protagonists in tumor evolution, resistance, and therapeutic vulnerability (Gulati et al., 2026; Arora et al., 2024; Wu et al., 2019).

The convergence of multi-omics profiling, single-cell genomics, AI-driven network modeling, and advanced liquid biopsy platforms has created an unprecedented technological infrastructure for interrogating GPCR signaling dynamics in real time, enabling the translation of biological complexity into actionable clinical intelligence. Complementary therapeutic modalities—biased ligands, PROTACs, CRISPR-based screening, RNA therapeutics, and digital twin-guided adaptive regimens—collectively expand the actionable GPCR target space beyond the reach of conventional oncology pharmacology (Hsu et al., 2025; Jiang et al., 2025).

Realizing the full potential of this framework will require concerted effort to address standardization gaps in liquid biopsy platforms, develop AI tools with prospective clinical validation, design adaptive trial architectures suited to dynamic biomarker-guided interventions, and dismantle access barriers that currently confine precision oncology to well-resourced healthcare systems. Meeting these challenges will unlock GPCR-centric precision oncology as a transformative force for improving durable survival across diverse cancer types, ultimately converting the adaptive resilience of tumors from an obstacle into an exploitable therapeutic vulnerability (AbdelHamid et al., 2026; Alum, 2025; Fountzilas et al., 2024).
