# Supplementary material for: GPCR signaling systems facilitate precision interventions for multi-oncology therapy
Source: Front Syst Biol. 2026 Sep 22;6:1940022. doi: 10.3389/fsysb.2026.1940022 (PMC13639398; doi:10.3389/fsysb.2026.1940022)
Supplement: Supplementary file 1 [file Table1.docx]

# Table 1. Conventional vs. GPCR-Guided Precision Oncology

| **Dimension** | **Conventional (Mutation/ctDNA-Centric)** | **GPCR-Guided (Expression/Liquid-Biopsy-Centric)** |
| --- | --- | --- |
| Primary analyte | ctDNA; tissue DNA sequencing | ctRNA, exosomal miRNA/protein, CTCs, complemented by cfDNA |
| Information captured | Somatic mutations, CNVs, MSI/TMB | Transcriptional, epigenetic, and signaling-state changes, including non-mutational resistance |
| Temporal resolution | Typically single time-point (diagnosis or progression) | Designed for serial, longitudinal sampling |
| Resistance mechanisms captured | Acquired resistance mutations | Additionally captures transcriptional upregulation, epigenetic reactivation, TME-driven adaptation |
| Clinical maturity | Established (e.g., Guardant360, FoundationOne Liquid CDx) | Emerging; no equivalent regulatory-approved comprehensive panel yet exists |
| Representative clinical validation | EGFR TKIs, BRAF/MEK inhibitors, PARP inhibitors | CXCR4-targeted PET imaging; early-phase CXCR4/Frizzled trials (Table S5) |
