# Supplementary material for: GPCR signaling systems facilitate precision interventions for multi-oncology therapy
Source: Front Syst Biol. 2026 Sep 22;6:1940022. doi: 10.3389/fsysb.2026.1940022 (PMC13639398; doi:10.3389/fsysb.2026.1940022)
Supplement: Supplementary file 2 [file Table2.docx]

# Table 2. Evidence-Tier Classification of Major Technologies and Modalities Discussed

| **Technology / Modality** | **Evidence Tier** | **Basis** |
| --- | --- | --- |
| CXCR4 small-molecule / peptide antagonism (e.g., motixafortide, plerixafor) | Clinical | Multiple completed/ongoing trials (Table S5); FDA-approved for stem-cell mobilization |
| CXCR4-targeted PET imaging | Clinical | In clinical use for hematologic malignancy/solid tumor imaging (Cheng et al., 2024) |
| Anti-Frizzled antibody (vantictumab) | Clinical (discontinued) | Phase I/Ib trials completed; development limited by bone-toxicity signal (Diamond et al., 2020) |
| FZD7 small-molecule inhibitors (e.g., cycloartobiloxanthone) | Preclinical | Multi-cohort in vitro/in vivo validation only (Sun et al., 2025) |
| GPCR-targeted PROTACs | Preclinical | No oncology trials identified to date (Saca et al., 2025) |
| GPCR-targeted RNA therapeutics (ASO/siRNA/CRISPR modulators) | Preclinical | Delivery/PK challenges remain unresolved (Yuan et al., 2024) |
| GPCR-directed CAR-T constructs | Preclinical / early discovery | AI-optimized antibody discovery stage (Liu et al., 2025) |
| DCyFIR / CRISPR multiplexed profiling | Preclinical (discovery platform) | Assay technology, not itself a therapeutic (Kapolka et al., 2020) |
| TMO-Net and related AI network models | Computational / predictive | No prospective clinical validation identified (Wang et al., 2024) |
| GPCR digital twins | Computational / predictive | Patient-cohort training, not prospectively validated (Boeringer et al., 2025) |
| Closed-loop AI-integrated monitoring platform (Section 6.3) | Theoretical / conceptual | Aspirational framework proposed by this review |
