# Supplementary material for: GPCR signaling systems facilitate precision interventions for multi-oncology therapy
Source: Front Syst Biol. 2026 Sep 22;6:1940022. doi: 10.3389/fsysb.2026.1940022 (PMC13639398; doi:10.3389/fsysb.2026.1940022)
Supplement: Supplementary file 3 [file Table3.docx]

# Table 3. Clinically Relevant GPCRs Across Cancer Types

| **GPCR** | **Cancer Type(s)** | **Associated Pathway / Role** | **Nature of Clinical Association** | **Key Source** |
| --- | --- | --- | --- | --- |
| CXCR4 | Breast, pancreatic, AML, myeloma, and >20 other cancers | CXCL12/CXCR4 axis; metastatic homing, immune evasion, BM niche retention | Prognostic-correlative; multiple therapeutic trials (see Table S5) | Furusato et al., 2010; Hamshaw et al., 2023 |
| GPRC5A (RAI3) | NSCLC; esophageal squamous cell carcinoma | Immune cell exclusion; pulmonary colonization of CTCs | Prognostic-correlative (survival association) | Lin et al., 2024; Zhou et al., 2024 |
| FZD7 | Glioblastoma; breast cancer (via pan-Frizzled targeting) | Wnt/β-catenin; EMT and mesenchymal transition; therapy resistance | Preclinical (glioblastoma); early clinical, discontinued (breast, vantictumab) | Dreyer et al., 2023; Diamond et al., 2020 |
| GPR65 / GPR4 / GPR68 | Pan-cancer (acidic tumor microenvironment) | Proton-sensing; pro-survival/pro-migratory signaling under acidosis | Preclinical / mechanistic | Ahn and Kaipparettu, 2025; Cornell et al., 2024 |
| GPR56 | Glioblastoma | Mesenchymal transition (single-cell multi-omics) | Preclinical | Ganesh et al., 2022 |
| Aminergic GPCRs (class-dependent) | Multiple (context-dependent) | β-arrestin-biased signaling; structure-guided ligand design | Preclinical / structural | McCorvy et al., 2018 |
