# Supplementary material for: GPCR signaling systems facilitate precision interventions for multi-oncology therapy
Source: Front Syst Biol. 2026 Sep 22;6:1940022. doi: 10.3389/fsysb.2026.1940022 (PMC13639398; doi:10.3389/fsysb.2026.1940022)
Supplement: Supplementary file 4 [file Table4.docx]

# Table 4. GPCR-Directed Therapeutic Modalities

| **Modality** | **Example GPCR Target(s)** | **Developmental Stage** | **Principal Limitations** |
| --- | --- | --- | --- |
| Biased ligands | Aminergic GPCRs; FZD7 | Preclinical to early clinical (target-dependent) | Bias can be assay- and system-dependent; translation to durable in vivo selectivity not fully established |
| PROTACs | Scaffolding/PPI-dependent GPCRs | Preclinical | Delivery, tissue selectivity, and degrader pharmacokinetics remain unresolved; no oncology trials identified |
| RNA therapeutics (ASO, siRNA, mRNA, CRISPR modulators) | Conserved GPCR signaling hubs | Preclinical | Delivery to solid tumors, off-target effects, and durability of knockdown remain limiting |
| CRISPR-based discovery platforms (e.g., DCyFIR) | Pathway-selective ligand discovery | Preclinical (discovery tool) | Accelerates hit-finding but does not itself constitute a therapeutic |
| GPCR-targeted CAR-T / antibodies | Novel CAR-T target GPCRs | Preclinical / early discovery | On-target, off-tumor toxicity risk given GPCR expression on normal tissue |
| Antibody-drug conjugates | Surface-expressed oncogenic GPCRs | Preclinical / early clinical (emerging class) | Payload toxicity and linker stability are unresolved for most GPCR targets |
| Small-molecule antagonists | CXCR4 (e.g., motixafortide, plerixafor), FZD7 (cycloartobiloxanthone) | Most clinically mature; several completed/ongoing trials | Chronic dosing toxicity (e.g., HSPC mobilization effects); receptor redundancy can blunt efficacy |
| Digital twin-guided regimen selection | Patient-specific signaling network | Computational / predictive; not prospectively validated | No completed prospective trial has yet tested a digital-twin-triggered treatment switch |
