# Supplementary material for: GPCR signaling systems facilitate precision interventions for multi-oncology therapy
Source: Front Syst Biol. 2026 Sep 22;6:1940022. doi: 10.3389/fsysb.2026.1940022 (PMC13639398; doi:10.3389/fsysb.2026.1940022)
Supplement: Supplementary file 5 [file Table5.docx]

# Table 5. Representative Registered Clinical Trials Targeting GPCRs in Oncology

| **Trial ID** | **Intervention** | **Cancer Type** | **Phase** | **Primary Endpoint** | **Status** |
| --- | --- | --- | --- | --- | --- |
| NCT02826486 (COMBAT trial) | Motixafortide (BL-8040, CXCR4 antagonist) + pembrolizumab ± chemotherapy | Metastatic pancreatic ductal adenocarcinoma | IIa | Objective response rate | Completed (Bockorny et al., 2020) |
| NCT02502968 | Motixafortide + high-dose cytarabine consolidation | Acute myeloid leukemia (first remission) | II (randomized, placebo-controlled) | Relapse-free survival | Completed |
| NCT01359657 | Ulocuplumab (anti-CXCR4 antibody) + lenalidomide/dexamethasone or bortezomib/dexamethasone | Relapsed/refractory multiple myeloma | Ib/II | Safety, response rate | Completed (Ghobrial et al., 2014) |
| NCT01973309 | Vantictumab (anti-Frizzled antibody, OMP-18R5) + paclitaxel | Locally recurrent/metastatic HER2-negative breast cancer | Ib | Safety / dose-limiting toxicity | Completed (Diamond et al., 2020) |
| NCT01345201 | Vantictumab (anti-Frizzled antibody, OMP-18R5), single agent | Advanced solid tumors | I | Safety / MTD | Completed |
| NCT05465590 | MB1707 (paclitaxel-conjugated CXCR4 antagonist) | Advanced solid tumors (breast, NSCLC, ovarian, pancreatic) | Early Phase 1 | Pharmacokinetics, safety | Withdrawn (sponsor decision) |
