# Supplementary material for: GPCR signaling systems facilitate precision interventions for multi-oncology therapy
Source: Front Syst Biol. 2026 Sep 22;6:1940022. doi: 10.3389/fsysb.2026.1940022 (PMC13639398; doi:10.3389/fsysb.2026.1940022)
Supplement: Supplementary file 6 [file Table6.docx]

**Table 6. GPCR-Directed Therapeutic Modalities in Oncology**

| **Modality** | **Mechanism** | **Example GPCR Target(s)** | **Key Advantage** | **Manuscript Section** |
| --- | --- | --- | --- | --- |
| Biased ligands | Selectively engage anti-tumor signaling arms (e.g., β-arrestin) while avoiding pro-tumorigenic G protein pathways | Aminergic GPCRs; FZD7 | Functional selectivity reduces on-target toxicity for chronic dosing | 4.4, 5.2 |
| PROTACs | Recruit E3 ubiquitin ligases to degrade overexpressed receptors rather than block signaling | Scaffolding/PPI-dependent GPCRs | Addresses receptors with no druggable orthosteric/allosteric pocket | 5.1 |
| RNA therapeutics (ASOs, siRNA, mRNA, CRISPR modulators) | Sequence-specific modulation of GPCR expression, independent of protein structure | Conserved GPCR signaling hubs across cancer types | Reaches "undruggable" GPCRs lacking small-molecule binding sites | 5.3 |
| CRISPR-based discovery platforms (e.g., DCyFIR) | Multiplexed, high-throughput profiling of receptor activation across signaling readouts | Pathway-selective ligand discovery | Accelerates identification of biased/selective compounds | 5.1 |
| GPCR-targeted CAR-T / antibodies | AI-optimized antibodies against surface-expressed GPCRs for immune cell engineering | Novel CAR-T target GPCRs | Extends immunotherapy to GPCR-overexpressing tumors | 2.3 |
| Small-molecule antagonists | Competitive/allosteric blockade of receptor-ligand engagement | CXCR4 (AMD3100), FZD7 (cycloartobiloxanthone) | Established pharmacology, clinically precedented | 4.1–4.3 |
| Digital twin-guided regimen selection | Computational modeling of patient-specific GPCR network behavior from longitudinal multi-omics data | Patient-specific signaling network | Enables adaptive, mid-treatment recalibration | 5.2, 6.3 |
